# Discharge Characteristics, Plasma Electrolytic Oxidation Mechanism and Properties of ZrO_2_ Membranes in K_2_ZrF_6_ Electrolyte

**DOI:** 10.3390/membranes12050516

**Published:** 2022-05-12

**Authors:** Li Wang, Wen Fu, Guangkun Yi, Ziyang Chen, Zhitin Gao, Qingyu Pan

**Affiliations:** 1 Key Laboratory of Inferior Crude Oil Processing of Guangdong Provincial Higher Education Institutes, Maoming 525000, China; wanglihaha@gdupt.edu.cn; 2 College of Chemical Engineering, Guangdong University of Petrochemical Technology, Maoming 525000, China; a2865976116@163.com (G.Y.); a2423350989@163.com (Z.C.); 3 College of Material Science, Guangdong University of Petrochemical Technology, Maoming 525000, China; a449192213@163.com (Z.G.); a390375448@163.com (Q.P.)

**Keywords:** plasma electrolytic oxidation, discharge, active species, mechanism, heat and mass transfer

## Abstract

ZrO_2_ was coated on AZ31 magnesium alloy substrate by plasma electrolytic oxidation with K_2_ZrF_6_ and NaH_2_PO_4_ electrolytes. The discharge characteristics and variation in active species during the plasma electrolytic oxidation (PEO) process were studied by optical emission spectroscopy. The surface morphology and element composition of the membranes were observed by scanning electron microscope. The ion transfer of the substrate was studied by atomic absorption spectroscopy. The phase composition and corrosion characteristics of the PEO membranes were examined with XRD and an electrochemical workstation, respectively. The heat and mass transfer models during the PEO process were introduced. The contributions of ions to the membranes and active species were also analyzed. The results indicated that the ion transfer at different stages exhibits different tendencies. At the first and transition stages, the migration resistance of the ions was low and increased gradually. At the initial discharge stage, the migration resistance was the highest because the highest membrane growth rate occurred at this stage. At the later discharge stage, the migration resistance tends to be stable, which is ascribed to a dynamic equilibrium PEO membrane growth rate. The intensity of active species is related to the energy state of the working electrode’s surface. The higher the energy, the greater the probability that the active species will be excited to generate energy level transitions, and the higher the plasma concentration.

## 1. Introduction

Plasma electrolytic oxidation (PEO) technology is an effective method for the surface modification of metallic materials. It can grow ceramic membranes on the substrate in situ due to the mutual effects of electrochemistry, plasma chemistry, and thermal chemistry. Because the chemical reaction happens between the interface of the substrate/electrolyte and the electrolyte/substrate, rather than by simple physical deposition, the membranes are strongly adhered to the substrate with different compositions, membrane thickness, colors, strength, hardness, friction property, photocatalytic activity, etc. [1,2,3,4], thereby strengthening the surface of the substrate. For example, the antibacterial effect and corrosion behavior could be improved in Ringer’s physiological solution when the PEO membranes are processed by adding nanoparticles of ZnO [3].

Some important features of the PEO process are the phenomena of fulmination, luminescence, discharge, and heat release that occur between the interfaces of substrates/electrolytes [5]. At different PEO stages, the continuously moving micro-discharge sparks with different shapes, colors, and numbers appear on the surface of the substrate. The plasma discharge plays a crucial role in the formation, structure distribution, and composition phase of the PEO membranes, which determines the thermodynamic process and chemical state changes on the surface of the substrate. These mechanisms are complicated, and are related to thermal chemistry, acoustics, plasma chemistry, electrochemistry, and so on [6,7,8,9,10].

The composition and characteristics of active species in the plasma field and their influence on the PEO process are important for understanding the mechanism of PEO. Therefore, analysis of the discharge active species in the plasma field and its thermodynamic state is of great significance.

The breakdown and discharge phenomena are other important features of PEO technology that have been studied by many researchers [6,7,8,9,10]. The PEO process is accompanied not only by the chemical reaction of the substrate, but also by the reaction of the heated substrate materials with oxygen before the breakdown of the dielectric barrier layer. The breakdown process of the dielectric barrier layer is complicated, involving the solid/liquid, liquid/gas, gas/liquid, and gas/solid interfaces. Heat and mass transfer between these interfaces and different types of reactions might be involved. The tools for analyzing these details are currently limited.

However, most research has focused on improving the performance and micro-structure of the membranes, such as the photocatalytic activity of the TiO_2_ membranes prepared by PEO [2], the antibacterial effect, corrosion behavior and thermal control of membranes on pure titanium substrate [3,6], the nanocrystalline and amorphous structures [4,9] on the PEO membranes, and so on. Marti-Calatayud [7] reported that anion-exchange membranes made from inexpensive ceramic materials were synthesized using a simple procedure based on incorporating particles of an ion exchanger into a host microporous structure. The hydrated zirconium dioxide was deposited in the porous network of the membranes by direct precipitation. It showed that the loading of hydrated ZrO_2_ particles improved the ion exchange capacity and induced anion-selective properties on the membranes. Dzyazko [9] studied the composite membranes obtained by modification of macroporous ceramics with hydrated zirconium dioxide and basic bismuth nitrate. This modification technique provided the formation of the two-component modifier directly in the macropores of the inorganic matrix.

What is the influence of the ions in the electrolyte on the heat and mass transfer between the interfaces? How do the ions affect the plasma field and active species? How are the PEO membranes formed? What is the relationship between the ions and the PEO membrane composition? Wail [11] used ab initio DFT (density functional theory) calculations to verify the experimental results and provide a theoretical understanding of the interfacial interactions and weak bonds arising in the self-assembly of organic molecules. The DFT calculations were also used to confirm the experimental results and deepen the understanding of the nature of the complex interactions in the self-assembly. They attempted the consecutive deposition of a two-dimensional supramolecular array on another to fabricate an organic layer and realize the growth mechanism on the underlying inorganic layer of distinct ordered layers, each of which is stabilized by in-plane noo-covalent bonds [12]. Yu [13] shows the Nyquist plots of the PEO coatings. In the frequency case of control by diffusion in the electrolyte or in a surface film or coating, an additional resistive element called Warbruge impedance was demonstrated. Mehri [14] explored the precise effect of colloidal TiO_2_ nanoparticles on the microstructure and properties of Al_2_O_3_-TiO_2_ composite coatings. They discussed two growth mechanisms of the PEO process. Up to now, little research has reported the influence of the ions in the electrolyte on the heat and mass transfer between the interfaces. Further studies on the composition of plasma active species and the characteristics of micro-discharges are necessary to answer these questions.

The fast-imaging digital camera, optical emission spectroscopy (OES) and scanning electron microscope were used to further investigate the discharge mechanism of the PEO process and the influence of the anion and cation in the electrolyte on the plasma field and the PEO membranes. The influence of cations and anions on ion transfer was derived. The heat and mass transfer models and PEO membranes’ growth mechanisms were also introduced. The sources, orbit transitions, and transition orders of the active species were also discussed.

## 2. Materials and Methods

### 2.1. PEO Membrane Preparation

The details of the PEO membrane preparation were reported in our previous studies [1,5]. The metal substrate was an AZ31 magnesium alloy. The power supply was a home-made DC power unit providing voltages of 0–1000 V and a current of 0–3 A. The mole concentration of K_2_ZrF_6_ was 0.025 mol/L. The mole ratio of K_2_ZrF_6_ to NaH_2_PO_4_ was 9:1 to ensure the pH of the electrolytes was around 4.5. The samples were treated under a voltage of 460–470 V for 30 min. The voltage and current were recorded with a data acquisition system. The sampling period of the data was 200 ms. The reagent is from Sinopharm Chemical Reagent Co., Ltd., Shanghai, China.

### 2.2. Evolution of the Surface of the Substrate

The evolution of bubbles and micro-discharges on the surface of the substrate was recorded by the digital camera’s fast imaging (D80, Nikon, Tokyo, Japan; effective pixels at 10 million and shutter at 1/350 s).

The details of the active species of the micro-discharges in the plasma field formed during the PEO process were captured by optical emission spectroscopy (OES), which has been reported in our previous studies [1,5]. Subsequently, the transition orbits of each special species at each wavelength were also confirmed according to this reference.

### 2.3. Test of the PEO Membranes and Electrolytes

AA-6800 atomic absorption spectrometry (AAS) was used to analyze the concentration of dissolved Mg^2+^ in electrolytes, comprising just the dissociative magnesium ions in the electrolyte. Samples were taken out after the experiments finished at every fixed voltage and reaction time. The electrolyte was mixed fully before sampling. Then the solution was diluted to certain concentrations and analyzed with AAS.

The micro-morphology of the PEO membranes was observed with a scanning electron microscope (SEM) (LEO 1530 VP, Japan) at different voltages. The elements and content of the membranes were obtained with the energy dispersive X-ray spectroscopy (EDX) affiliated with SEM.

The phase composition of the PEO membranes was analyzed by (Dmax-3A, Netherlands) (X-ray Diffraction, XRD).

Corrosion resistance of the oxide membranes was tested by potentiodynamic polarization tests using an Autolab system (Autolab, Luzern, Switzerland) in 3.5 wt.% NaCl solution at a temperature of between 30 and 33 °C. All samples were sealed with epoxy resin, and the tested areas were 0.5 cm^2^. A platinum plate and a saturated calomel electrode (SCE) were used as the counter and reference electrode, respectively. The scanning rate was 1 mV/s from −1.6 V to −0.9 V versus the reference electrode.

## 3. Results and Discussion

### 3.1. Discharge Characteristics and Surface Variation during the PEO Process

**Figure 1:**
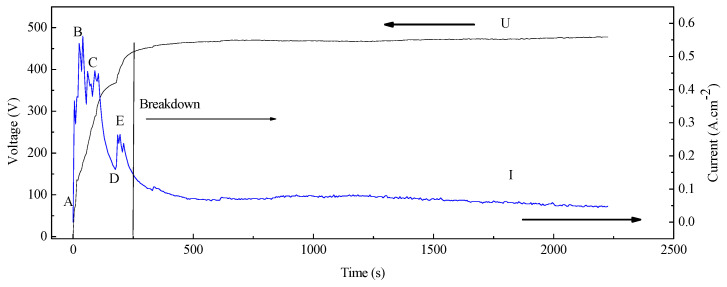
The discharge characteristics of K_2_ZrF_6_ electrolyte during the PEO process.

**Figure 2:**
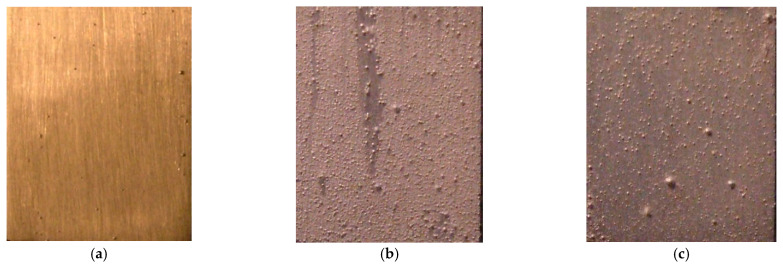

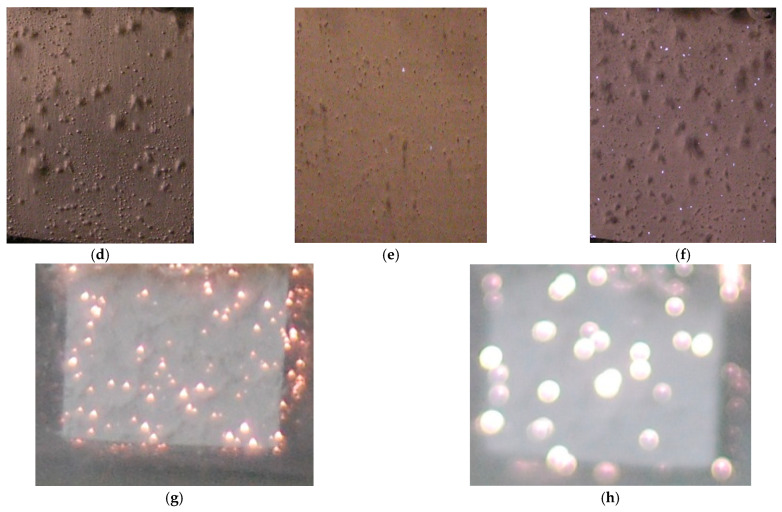
The bubbles and discharge sparks’ evolution on the surface of the working electrode during the different PEO processes at different voltage and time (**a**) 400 V-30 s; (**b**) 100 V-30 s; (**c**) 400 V-10 s; (**d**) 400 V-30 s; (**e**) 420 V-30 s; (**f**) 466 V-1 min; (**g**) 466 V-5 min; (**h**) 466 V-10 min.

Voltage-time and current-time curves of the PEO process with K_2_ZrF_6_ electrolyte are shown in Figure 1. The evolution of bubbles and micro-discharges on the surface of the substrate at different voltages and times is shown in Figure 2. In the first stage, named the conventional anodic oxidation stage, the current density increased sharply with the voltage in a short time (Figure 1 (AB region)). The substrate surface began to lose the metallic luster and emit light. The bubbles also quickly aggregated on the metal surface (Figure 2a,b). The second stage was the transition stage. When the voltage was increased, the solution around the substrate was heated by the Joule heating effect, reaching the vaporization temperature. The saturation temperature around the substrate began to initiate the nucleation and formation of the bubbles (Figure 2c,d), which started from Point B (Figure 1). At Point B, the current was at its maximum, so the energy on the surface of the substrate was at its maximum. Energy aggregation is a necessary condition for the formation of a gas envelope. When it reached Point C, the substrate was completely covered by the gas envelope composed of a group of bubbles (Figure 2d). Thus, the substrate and the electrolyte were completely isolated by the gas envelope. Once the gas envelope was formed (Figure 2c), its conductivity and resistance increased, resulting in a decrease in the current density (Figure 1 (BC region)). As the voltage continued to increase, the amount and thickness of the gas envelope increased continuously (Figure 2d), and the resistance of the gas-liquid system increased significantly; therefore, a sharp decrease in the current density occurred (Figure 1 (CD region)). At Point D (Figure 1), the voltage reached the breakdown level. The energy aggregation was enough to break down the gas envelope layer, followed by the dielectric barrier layer. At the same time, collapse and ionization occurred in the gas bubbles, initiating the continuous plasma micro-discharge inside the bubbles, which triggered the inelastic collision and electron avalanche; then the electrolyte entered the discharge stage (Figure 1 (DE region), Figure 2e). The breakdown initially occurred at the weakest dielectric layer point, where the dopants and defects, termed “flaws” (such as micro-fissures, cracks, local regions of different compositions, and impurities), were located [15,16,17,18,19,20].

When the voltage continued to increase above the breakdown down Point D (Figure 1), the current density continued to increase, which may have been due to the increased surface area treated by the plasma micro-discharge (Figure 2e,f). After Point E (Figure 1), it entered a stable plasma micro-discharge stage (Figure 2g,h). During this stage, as the membrane layer gradually thickened, a higher energy density was required to pass through the membrane layer (dielectric barrier layer) and the gas envelope, so the spark energy gathered gradually. In this case, the energy concentrated on the weakest part of the membrane layer, continuing to break down the dielectric barrier layer. Then the new plasma micro-discharge was generated, so at this stage, the current density decreased gradually after Point E (Figure 1). After the dielectric barrier layer was broken down, where the place would be filled by the molten oxide again as quickly as possible, the dense dielectric barrier layer was formed again, after which the discharge continued at the weakest place. After Point E, the sparks’ density decreased, their size increased, and their color changed (Figure 2g,h), which could be mainly attributed to the thickness of the membrane growth, the surface energy of the membrane, and the number of active species in the plasma field. The change in spark color was related to the metal ion flame reaction. The flame color reaction of Na is yellow, the flame reaction of K is purple, and that of Mg and Al is flameless. The transition of Na from 3p orbit to 3s orbit requires an excitation energy of 2.10 eV, and the excitation energy required for K from 4s orbit to 3d orbit is 2.56 eV. The excitation energy required for Mg is 7.65 eV. The micro-discharge sparks’ color was mainly yellow at the initial discharge stage (Figure 2g), because the excitation of Na needed less energy. The greater the surface energy of the substrate on some dopants and defect areas at the later discharge stage, the greater the probability that K, Al, Mg would be excited, changing the color of micro-discharge sparks to purple and white. Therefore, a micro-discharge spark’s color changes with the surface energy of the substrate.

### 3.2. Variation in the Spectroscopy during the PEO Process

**Figure 3:**
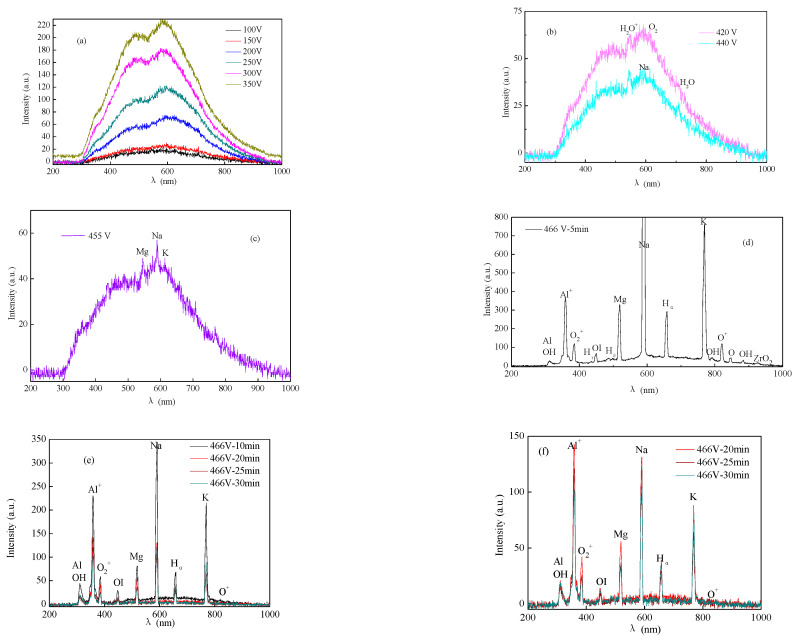
The light emission spectra in K_2_ZrF_6_/NaH_2_PO_4_ electrolytes during the PEO process at different voltage and time (**a**) 100 V, 150 V, 200 V, 250 V, 300 V, 350 V; (**b**) 420 V, 440 V; (**c**) 455 V; (**d**) 466 V-5 min; (**e**) 466 V-10 min, 466 V-20 min, 466 V-25 min, 466 V-30 min; (**f**) 466 V-20 min, 466 V-25 min, 466 V-30 min.

**Figure 4:**
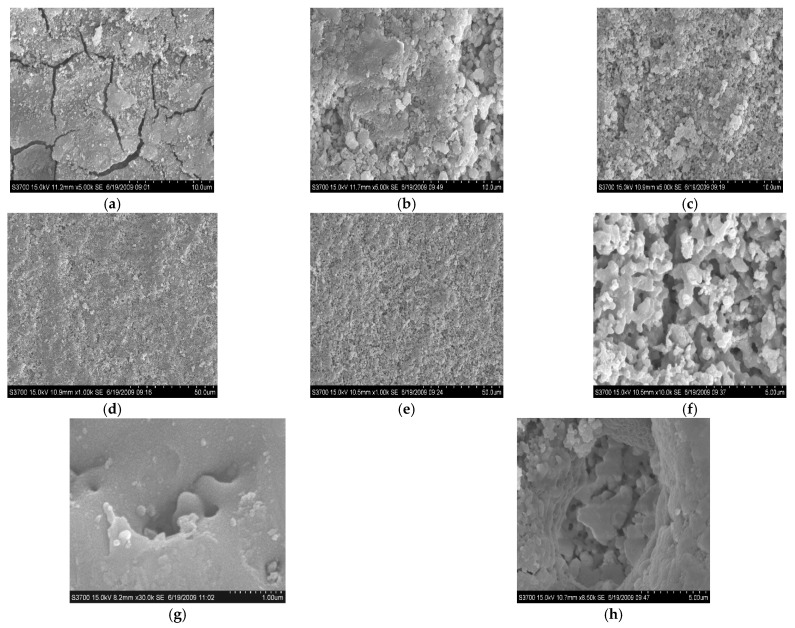
The surface morphologies of the membranes formed in K_2_ZrF_6_ and NaH_2_PO_4_ electrolytes at different voltage and time (**a**) 100 V-30 s; (**b**) 200 V-30 s; (**c**) 300 V-30 s; (**d**) 400 V-30 s (**e**) 420 V-30 s; (**f**) 466 V-10 min; (**g**) 466 V-10 min; (**h**) 466 V-10 min.

Figure 3 shows the variation in the spectroscopy during the PEO process. Figure 4 shows the micro-morphology of the membranes at different voltages. At the conventional anodic oxidation stage, there were no sharp peaks (Figure 3a) on the spectra because there were no micro-discharges at this stage. As discussed in our previous study [1,5] and by other reports [16,17,18,19,20], the light emission at this stage could be attributed to the phenomena of galvanoluminescence (GL) and electroluminescence. Stojadinovic and Shimizu [16,17,18,19,20] reported that the flaws in the oxide membranes resulted in light emission. The flaws may result from impurities on the substrate surface or on the dielectric barrier layer. As shown in Figure 4a, many cracks were exhibited on the surface of the membranes during the initial conventional anodic oxidation stage (100 V-30 s). The cracks in membranes decreased as the voltage increased, but the surface was very rough, and the different oxides were distributed unevenly on the surface of the substrate (Figure 4b). The surface morphology of the membranes tended to be relatively uniform (Figure 4b–d) as the voltage increased from 200 V to 300 V, or even to 400 V; that is, the dielectric barrier layer tended to be more uniform, although the intensity of the GL was suggested to be roughly proportional to the quantity of flaws in the oxide membranes [16,17,18,19,20]. At the conventional anodic oxidation stage (Figure 3a), the light intensity gradually increased with the increase in the voltage, consistent with the results showing that the current density increased with the voltage because the dielectric barrier layer was not uniform and dense. Once the flaws were formed under the oxide membranes, higher conductivity in the oxide membranes corresponded to higher current density. Thus, the current density could represent the flaws of the oxide membranes to some extent.

At the transition stage, the collected weak spectral signals were mainly from H_2_O^+^ (549.3 nm), O_2_ (628.0 nm), and H_2_O (719.2 nm), which further proves that the breakdown first occurred in the bubbles of the gas envelope. As shown in Figure 3b, the light intensities at 420 and 440 V were 63 and 43 a. u., respectively, which was 225 a. u. at 350 V. Because the relatively uniform gas envelope and dielectric barrier layer were formed, the aggregated energy was used to break down the gas envelope, followed by the dielectric barrier layer. The collapse and ionization of the bubble layer occurred at the transition stage. Previous studies [1,5,21] have reported that the current density at the transition stage decreased sharply with increasing voltage, as shown in Figure 1 (BD region). The current density can reflect the energy on the surface of the substrate. The decrease in light intensity should be related to the decrease in energy on the whole substrate surface [1,5,21]. The energy might be converted into heat or other forms, so the light intensity decreases sharply at the transition stage.

Figure 3c presents the OES spectra obtained from the K_2_ZrF_6_ electrolyte at the plasma discharge stage. As shown in Figure 3c, the active species of Na (589.01 nm), K (769.50 nm), and Mg (519.40 nm) appeared, Na and K were from the metal substrate, but K was from the electrolyte. As discussed in our previous studies [5], the order of the excited active plasma species depends on the excitation energy of the species. At the discharge stage, the energy in the plasma field increases with the increase in the voltage. The metal elements were excited first because their excitation energy was lower than that of the H or O elements. After the metal elements were excited, all active species were excited (Figure 3c,d), including O (847.03 nm), H (309.30 nm), OH (882.94 nm), Na (589.01 nm), K (769.90 nm), O_2_ (759.37 nm), O_2_^+^ (383.05 nm), and OI (448.72 nm), as detected in the OES spectra. These atoms and molecules were mainly from the substrate and gases produced on the surface of the substrate (such as H_2_O or O_2_, or even the ionization of H_2_O and O_2_). At the initial discharge stage, the voltage increased from 455 to 466 V, and the spectra intensity increased from 60 to over 800 a. u. At the later discharge stage, because the sparks’ density decreased and the size increased owing to the increasing thickness of the membranes and the decreasing energy on the substrate, the spectra intensity decreased gradually (Figure 3e).As the treated time changed from 466 V-5 min to 466 V-10 min, the highest spectra intensity decreased from over 800 to 350 a. u.; at the same time, some active species that needed higher energy to be excited were eliminated, such as H_γ_ and H_β,_ as shown in Figure 3e. After 466 V-20 min (Figure 3f), the spectra intensity tended to gradually stabilize, and the highest intensity was around 130–140 a. u. These results are consistent with the results of the variation of the current density, which decreased to a stable value gradually at a later discharge stage (as shown in Figure 1).

As discussed above, the variation tendency of the OES spectra was quite consistent with the change in the current density, which represents the energy state of the entire substrate to some extent.

Some micro-discharge channels could be seen after the samples were treated with the micro-discharge for some time, as shown in Figure 4e; the melting and sintering morphology is shown in Figure 4f. When the discharge channel morphology magnified from 30 K (Figure 4g) to 50 K (Figure 4h), the different layer extended to the outside. In addition, there were many different inner discharge channels distributed in the outside discharge channel, which proves that the PEO membranes grow layer by layer from the inner layer to the outer layer.

### 3.3. The Spectra Intensity Variation of Each Active Species

**Figure 5:**
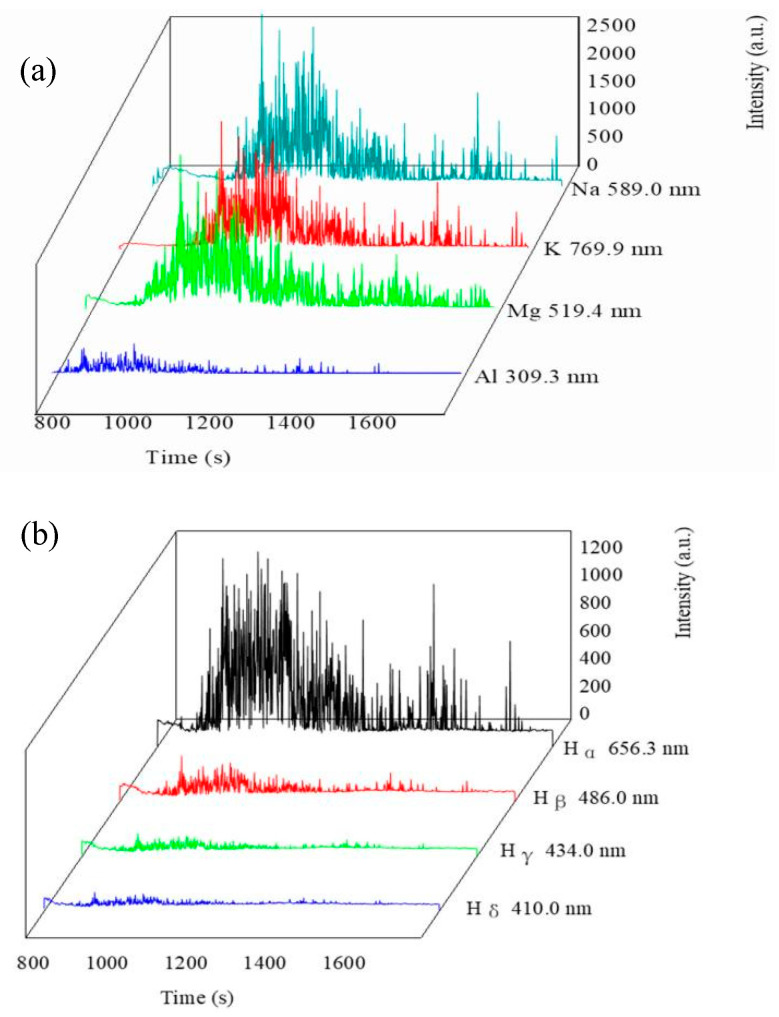

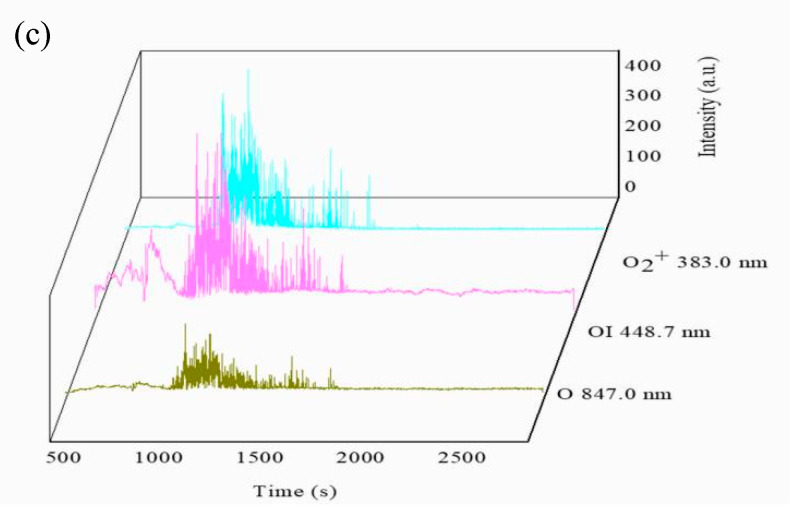
The intensity variation of each special plasma species with the time. (**a**) Metal elements; (**b**) Balmer lines of hydrogen; (**c**) oxygen elements.

Figure 5 shows the relationship of the single active species with the changing time. A similar oscillation trend in each active species is presented. A higher plasma concentration in the same active species corresponded to a stronger acquired spectral line intensity. According to the results discussed above (Section 3.1 and Section 3.2), first, the oscillation trend of each single active species with the changing time was related to the characteristics of the discharge sparks. Because the discharge sparks continuously hopped on the surface of the substrate during the PEO process, but the probe of the OES was fixed at one motionless place, the intensity of the spectra of each collected active species was sometimes strong and sometimes weak. This is one reason why the spectra exhibited the oscillation trend. Second, the oscillation trend was also related to the nature of the active species in the plasma field. The survival time of active species is very short. Shimizu [20] reported that the life of the active species was about 0.75 ms, and the active species quenched and regenerated repeatedly. This is another reason for the oscillation trend in the spectrum. Third, the spectra intensity oscillation was also related to the energy state of the substrate. As discussed above in Section 3.1, the current density could represent the energy state of the substrate to some extent. The variation trend of the current density also oscillated, which was related to the evolution characteristics of the substrate. During the period from 1000 to 1200 s, called the initial discharge stage, the spectral intensity of active species tended to be relatively stable, because at this stage, the micro-discharge spark was small, so the intensity of the spectra was weak and relatively stable. However, at the later discharge stage, the spectra intensity decreased gradually, because the PEO membranes gradually thickened, requiring more energy to aggregate enough to break down the dielectric barrier layer at the weakest point of the PEO membrane layer. The energy would transfer to other forms, so the spectra intensity at the later discharge stage was weaker compared with the spectra intensity at the initial discharge stage. These results further prove that the higher the energy of the entire substrate, the more the active species that would be excited in the plasma field. Conversely, the lower the energy of the substrate, the fewer the active species that would be excited. The oscillating trend of the spectral intensity indicated that the local concentration of the active species at the surface of the substrate was constantly changing, because the discharge spark was instantaneously quenched and regenerated.

As shown in Figure 5, it can be seen that at the discharge stage, the highest intensity of active species (Na, K, Mg, Al) excited from the metal elements was between 1500–2500 a. u. The highest intensity of the active species (H_α_, H_β_, H_γ_, H_δ_) was between 500–1000 a. u., while the highest intensity of active species (O_2_^+^, OI, O) was between 100–300 a. u. On the one hand, the intensity differences in these species may have been due to the fact that the active species excited by the metal elements had higher concentrations in the plasma field during the discharge stage, because the minimum excitation energy was required for the metal elements, followed by the H and O elements. On the other hand, it may be related to the energy state of each of the active species. The higher the energy the active species observed, the stronger was the intensity of the spectra. The excitation level transitions and energy requirements of each active species are shown in Table 1.

### 3.4. The Temperature of the Active Species

The temperature level of the micro-discharge in the plasma field was a very important parameter. As calculated in our previous study [5], the calculated electron temperature of hydrogen was 6 × 10^3^ and 3 × 10^4^ K, according to the Balmer hydrogen lines (H_α_, H_β_, H_γ_). The high-temperature environment provided the possibility for PEO ceramic membrane melting and sintering.

### 3.5. Ion Transfer

#### 3.5.1. The Ion Migration of the Substrate

**Figure 6:**
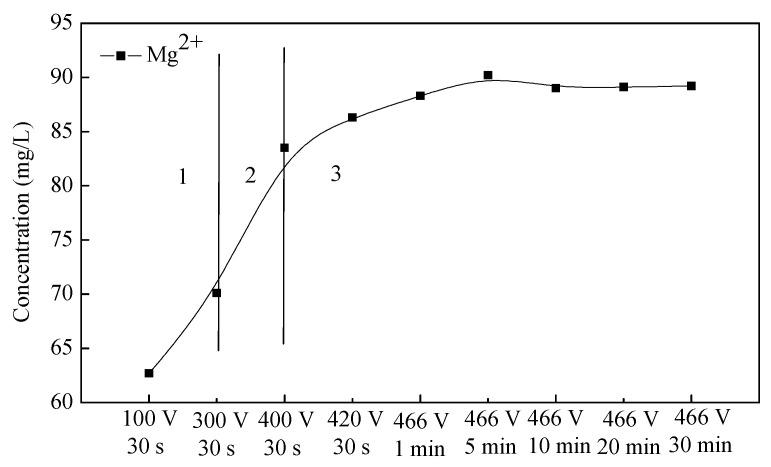
The changing curve of the amount of the dissolved Mg in the electrolyte during the PEO process.

Figure 6 shows the concentration distribution of Mg^2+^ from the substrate in the electrolyte. During the conventional anodic oxidation reaction, the magnesium ions were eluted, and at this stage, Mg^2+^ migrated to the electrolyte in a large amount, from 0 to 68 mg/L. These results also prove that at this stage, the growth rate of the membrane was slow, and the substrate was more susceptible to ionization. Furthermore, the migration resistance was low, so it was easier for magnesium ions to migrate from the substrate to the membrane/electrolyte interfaces. At the transition stage, the amount of Mg^2+^ migrated from the substrate increased rapidly from 68 to 83 mg/L when the voltage was increased from 300 to 400 V. Additionally, at the transition stage, the gas envelope was broken down, followed by the breakdown of the dielectric barrier layer, so the migration resistance was also low at this stage. Therefore, the dissolution rate of the magnesium ions was also larger at the transition stage. The migrated Mg^2+^ continuously increased at the initial discharge stage from 83 to 89 mg/L (before 466 V-5 min), because at this stage, the large number of discharge channels made it easy for Mg^2+^ to migrate into the electrolyte. At the later discharge stage (after 466 V-5 min), the variation trend of migrated Mg^2+^ tended to be relatively stable, with the concentration remaining between 88–89 mg/L. Because the membrane’s thickness increased gradually at the later discharge stage, as discussed above in Section 3.1, the number of micro-discharge sparks decreased, which resulted in the decrease in discharge channels. Therefore, the amount of migrated Mg^2+^ tended to be stable at the later discharge stage.

Under the electric field function, Mg^2+^ was produced by the anodic reaction of the substrate (reaction (1)). The cathodic reaction is shown in reaction (2).

(1) $$Mg\to Mg^{2+}+2e^{-}$$

(2) $$4OH^{-}\to O_{{}^{2}}+2H_{2}O+4e^{-}$$

#### 3.5.2. The Ion Migration of the Electrolyte

**Figure 7:**
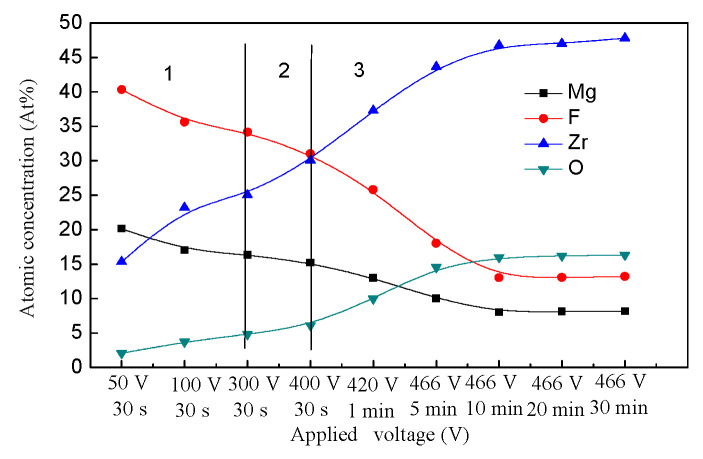
Qualitative analysis of element concentration in the oxide membranes formed at different PEO reaction stages.

The ion migration between the electrolyte/membrane interfaces was different because of the different reactions, the Joule heat, and the membrane thickness. To some extent, the composition of the membranes formed at different voltages during the PEO process reflects the ion migration between the electrolyte/membrane interfaces. The composition variation of the membranes is shown in Figure 7. As shown in Figure 7, the membranes were mainly composed of Mg, F, Zr and O elements. The element Mg came from the substrate and migrated into the electrolyte. Zr and F elements were derived from ZrF_6_^2−^ of the K_2_ZrF_6_ electrolyte. The source of O elements was complicated; they might come from the OH^−^ or from the O_2_ dissolved in the electrolyte during the PEO process, but the OH^−^ and O_2_ were from the water ionization. At the conventional oxidation stage, the Mg^2+^ was mainly from the anodic oxidation, and then migrated into the electrolyte. The ZrF_6_^2−^ was also ionized, and then migrated to the electrolyte/substrate interfaces to react with the substrate at the same time. At the transition stage, the inelastic collision and electron avalanche occurred, which was another way for the Mg^2+^ and F^−^ to migrate at the gas/electrolyte and substrate/gas interfaces aside from the electrode reaction. At the discharge stage, the micro-discharge channels were the main way for the ions to migrate between the gas/electrolyte and substrate/gas interfaces.

As shown in Figure 7, the element concentration of membranes formed during the PEO process presents different trends at different stages. At the traditional oxidation stage, the element content of Mg slowly decreased, while that of O and Zr slowly increased. The Mg content was about 40% at 50 V-30 s. The reason for this is that the substrate dissolved more magnesium ions in the initial oxidation stage by anodic oxidation, and they migrated to the substrate/electrolyte interfaces with the low resistance, and then participated in the PEO reactions (as shown in reactions (3) and (4)). Therefore, at the conventional anodic oxidation stage, the Mg content of the membrane was high, and at this stage, the ZrF_6_^2−^ and OH^−^ also migrated to the interfaces of the substrate/electrolyte to participate in the membrane-formation reaction. Under the high-energy conditions in the plasma field, the ZrF_6_^2^^−^ was dissociated into Zr^4+^ and F^−^, and Zr^4+^ underwent weak hydrolysis and ionization in water (as shown in reactions (5)). Ionized OH^−^ would accelerate the ionization of Zr^4+^, leading to the deposition of Zr(OH)_4_ (as shown in reactions (6)). Thus, the content of Zr and O increased at this stage. At the transition stage, the content of Mg and F still decreased, while that of Zr and O increased gradually. At the initial discharge stage (before 466 V-5 min), the content of Mg and F decreased sharply, indicating that the membranes’ growth rate was much higher at this stage. The membranes’ increased growth rate resulted in the increased resistance of the Mg and F ions migrated to the substrate/electrolyte interfaces. In addition, the content of O and Zr elements increased sharply, which may be attributed to the higher concentration of ZrF_6_^2−^ and OH^-^ in the electrolyte. After the gas envelope was broken down, ZrF_6_^2−^ and OH^-^ migrated to the electrolyte/substrate interfaces to participate in the PEO membrane reactions (as shown in reactions (5) and (6)). At the later discharge stage (after 466 V-5 min), the variation trends of Mg, F, Zr, and O contents became smaller and smaller, which indicated that the ion migration between the substrate/electrolyte and electrolyte/substrate interfaces had reached a dynamic equilibrium. That is to say, the PEO membranes’ growth rate also reached a dynamic equilibrium at this stage.

It could be concluded that at the conventional oxidation stage and transition stage, the migration resistance of the ions was low and increased gradually. At the initial discharge stage, the migration resistance was the highest because of the highest membrane growth rate. At the later discharge stage, the migration resistance tended to be stable with a dynamic equilibrium PEO membrane growth rate. Furthermore, the cations played an important role in the membranes’ composition.

(3) $$Mg\to Mg^{2+}+2e^{-}$$

(4) $$Mg^{2+}+2F^{-}\to MgF_{2}$$

(5) $$Zr^{4+}+4H_{2}O\to Zr{\left(OH\right)}_{4}↓+4H^{+}$$

(6) $$Zr{\left(OH\right)}_{4}\to ZrO_{2}+2H_{2}O$$

### 3.6. PEO Mechanism

#### 3.6.1. The Heat- and Mass-Transfer Models during the PEO Process

**Figure 8:**
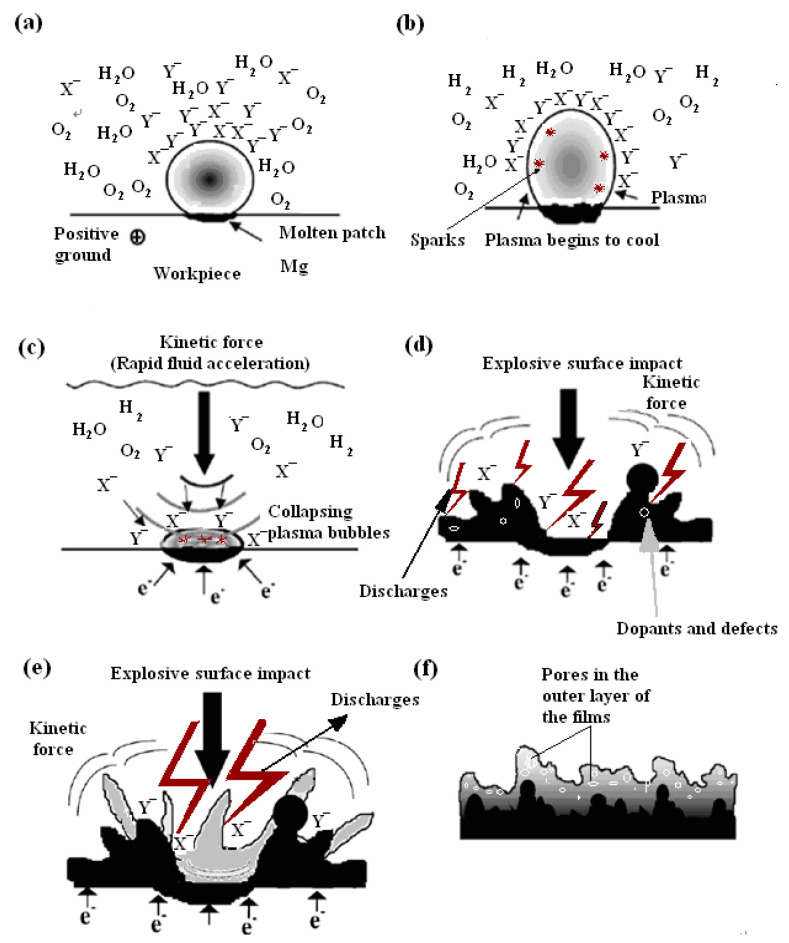
The heat and mass transfer models during the PEO process. X^−^, Y^−^—represents anions; e^•^—represents electrons. (**a**) the conventional anodic oxidation stage (**b**,**c**) the transition stage (**d**–**f**) the discharge stage.

There are many complicated chemical processes during the PEO process, such as thermo-chemistry, plasma chemistry, and electrochemistry, etc. There are also heat- and mass-transfer behaviors between the gas/liquid, gas/solid and liquid/solid interfaces. Based on the data discussed above, the simplified heat and mass transfer models were proposed to directly demonstrate the PEO mechanism, which is shown in Figure 8.

At the conventional anodic oxidation stage (Figure 8a), the surface of the substrate reacted with the electrolyte, and the gases (O_2_) were continuously generated on the substrate. When the energy of the surface of the whole substrate reached a certain value, water vapor (H_2_O) was also generated on the surface of the substrate. At this stage, the anions (X^−^ or Y^−^) in the electrolyte were also aggregated toward the surface of the substrate by the function of electric field. When the applied voltage reached a certain value, under the function of the thermal radiation, the surface of the substrate emitted weak light. At the transition stage (Figure 8b), the surface of the substrate was surrounded by a continuous gas envelope with insulation properties, and the stable plasma region formed. Under the function of the high pressure, the anions continuously accumulated on the surface of the substrate and were distributed on the surface of the bubble layer. Therefore, many local micro-domain strong electric fields were formed between the anode electrode surface and the negative charges. It was reported that this kind of electric field strength could reach 10^6^–10^8^ V/m or higher [25]. When this high electric field was formed, the gases in the bubbles were ionized (such as O_2_ or H_2_O, etc.). H_2_O would be ionized to produce H_2_ and O_2_; O_2_ would be further dissociated to O, O_2_^+^ or OI; H_2_ would be further dissociated to produce Balmer line H). The breakdown of the gas envelope occurred at this time (Figure 8b), resulting in the generation of electrons and active species. These dissociated high-temperature bubbles were surrounded by the cold electrolyte, resulting in the cooling of the plasma bubbles. Finally, the bubbles burst on the surface of substrate (Figure 8c) in a process known as “cavitation collapse” [26,27]. The formed dielectric barrier layer also exhibited an insulating property. When enough energy and electron avalanche were present to break down the dielectric barrier layer, the discharge occurred (Figure 8d). At the discharge stage (Figure 8d,e), the lifetime of the micro-discharge spark was very short (less than 7.5 ms), so the single micro-discharge spark was presented discontinuously, as shown in Figure 2g,h, and the substrate was surrounded by a large number of intermittent micro-discharge sparks rather than continuous micro-discharge sparks.

There were two cases of bubbles cracking on the surface of the substrate. First, the anions accumulated on the surface of the substrate, which was similar to the electron avalanche process, and then the collapse and ionization in the gas bubbles occurred, initiating the continuous plasma micro-discharge inside the bubbles, which triggered the inelastic collision and electron avalanche. Second, the energy stored in the bubbles was released into the gas envelop layer when the bubbles burst. At this time, the kinetic energy was transferred from the liquid layer to the surface of the substrate. This energy could be very high, similar to that of the cavitation (having a pressure of a few hundred MPa or more). The anions were accelerated by the holes created by the ruptured bubbles and moved to the surface of the substrate. This was the kinetic energy transfer (Figure 8e). The movement of ions was mainly due to the acceleration of ions generated by the function of the plasma field and the adsorption and migration of ion bubbles triggered by the bubble collapse. The migration of these two modes eliminated the diffusion of the phase boundary layer. The diffusion of the phase boundary layer was ubiquitous in the traditional electroplating process [28]. The PEO system was a dynamic system in which the high-speed movement of the stirring electrolyte caused the rapid migration of ions to reach the gas envelope for replenishment. As discussed in our previous studies [1,5,21], although the anion does not contribute to the composition of the plasma active species, it plays an important role in the charge balance of the entire system and the composition of the membrane layer. The localized high temperature in the plasma field resulted in the localized melting and sintering of the membrane layer. The surface of these membrane layers was quenched by the surrounding electrolyte after the bubbles in the plasma field burst, forming a unique micro-structure similar to the ceramic morphology. Many studies [3,10] have demonstrated that membranes are made up of three layers: a loose outer layer, a dense transition layer, and an inner layer (Figure 8f) [2,29].

#### 3.6.2. PEO Membrane Growth Mechanism

**Figure 9:**
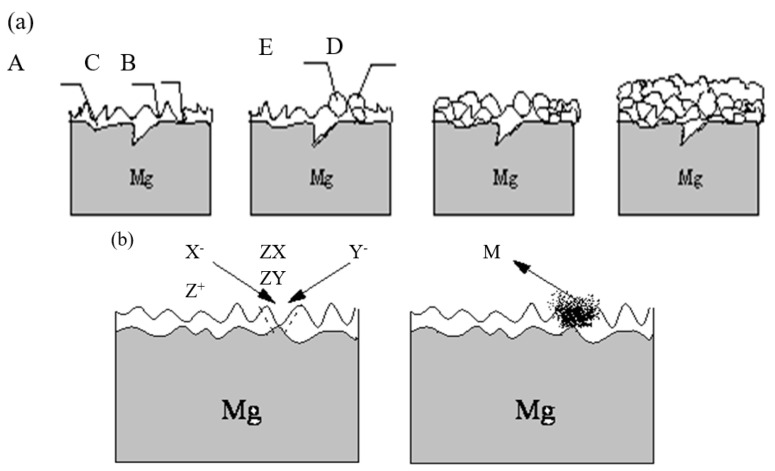
The schematic diagrams of the cross-sections of the molten ceramic membranes. (**a**) A—the surface of the pre-prepared membrane layer; B—the weakest place of the preprepared membrane layer; C—the relatively weak place of the preprepared membrane layer; D—the particles deposited on the weakest place of the membrane layer; E—the particles deposited on the relatively weak place of the membrane layer. (**b**) M—the particles; X^−^, Y^−^—the anions in the electrolyte; Z^+^—the cations in the electrolyte; ZX, ZY—the oxide particles suspended in the electrolyte.

Combining the results discussed in Section 3.1 relating to the micro-structure surface morphologies in Figure 4 and the heat- and mass-transfer models in Section 3.6.1, the PEO membrane growth model was shown in Figure 9. When the dielectric barrier layer was formed on the surface of the substrate, there were many flaws on or in the dielectric barrier layer. Following breakdown of the gas envelope, the breakdown occurred first on the weakest point of the dielectric barrier layer (Figure 9a-B), followed by the relatively weak point of the dielectric barrier layer (Figure 9a-C)). The breakdown lasted for a very short time, and was followed by a large number of micro-discharge sparks. The melting particles were deposited on the discharge places as a result of the micro-discharge sparks and mass transfer (Figure 9a-D/E)). This process happened repeatedly. Then the membranes gradually thickened. When the discharge occurred, the anions and cations migrated into the electrolyte/substrate interfaces via the discharge channels, followed by the oxide particles formed and deposited layer by layer on the surface of the substrate, which sintered and melted via the high-temperature plasma field (Figure 9b).

#### 3.6.3. SEM of Cross Section

**Figure 10:**
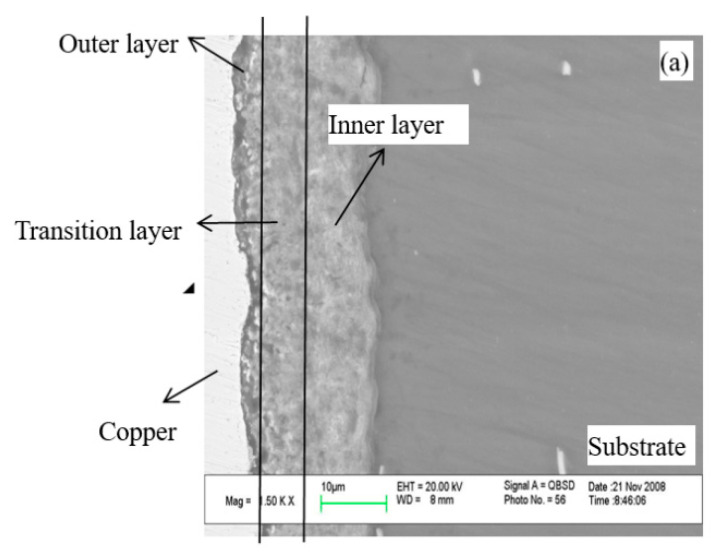

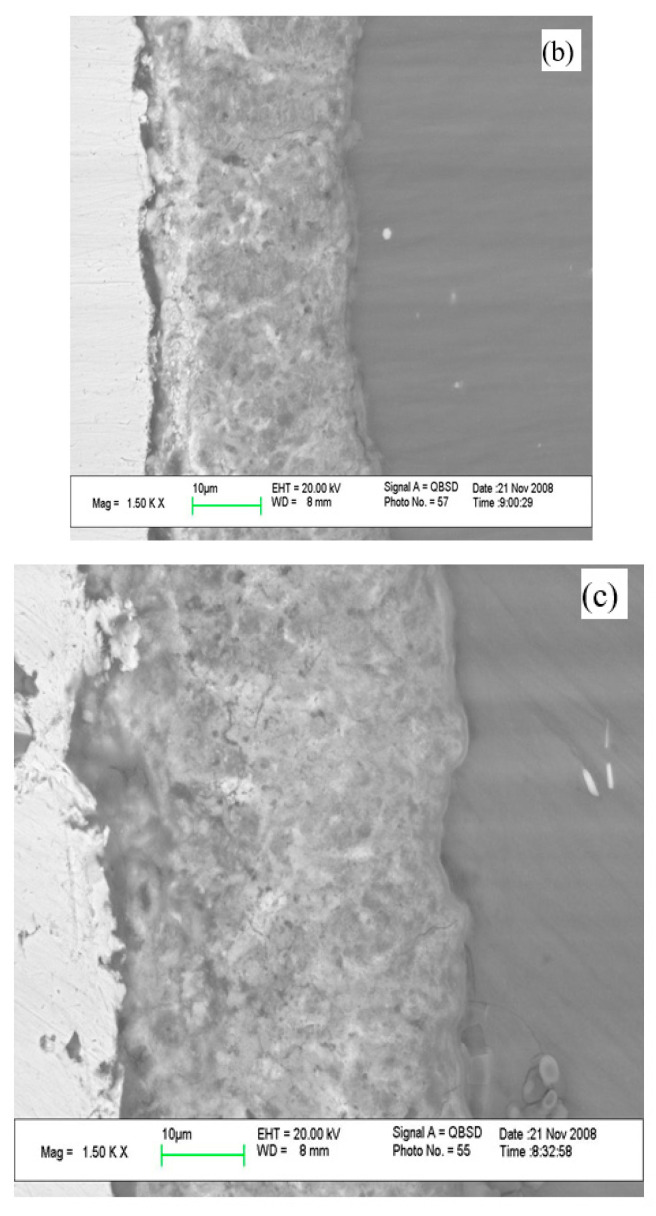
The SEM cross-sections of the membranes and. (**a**) at the transition stage 300 V-30 s; (**b**) at the earlier discharge stage 420 V-30 s; (**c**) at the later discharge stage 466 V-10 min.

SEM of cross sections at the transition stage and the discharge stage is shown in Figure 10. The film could be divided into three layers. These were the outer layer, the transition layer, and the inner layer. The films adhered well to the substrate. The outer layer was loose, and the transition and the inner layer were compact and could not be distinguished. What is more, the oxide particles were formed and deposited on the surface of the substrate layer by layer (Figure 10b,c). The sintering and melting of these oxide particles occurred in the high-temperature plasma field. The membranes grew thicker and thicker during the transition stage (Figure 10a) and discharge stage (Figure 10b,c).

### 3.7. PEO Membrane Properties

#### 3.7.1. Crystal Structure of PEO Membranes

**Figure 11:**
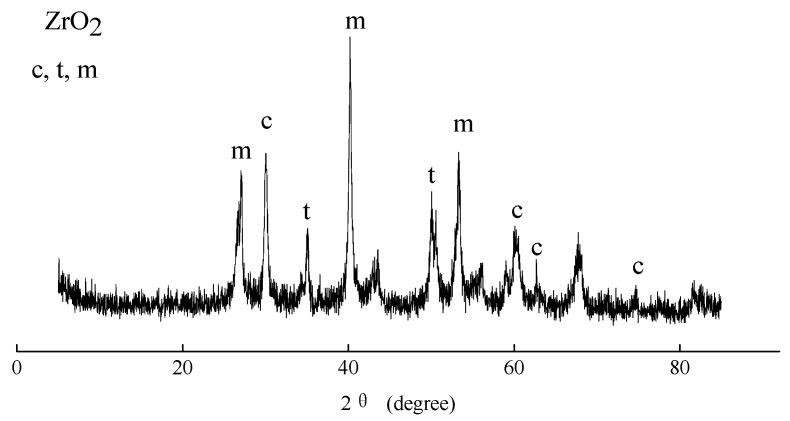
The phase compositions of the ZrO_2_ ceramic membranes.

Figure 11 shows the crystal structure of the ZrO_2_ ceramic membranes. It can be seen that the PEO ceramic membrane was mainly composed of ZrO_2_ and a little MgF_2_. ZrO_2_ has three different crystal phases: cubic crystal phase (c-ZrO_2_), tetragonal crystal phase (t-ZrO_2_), and monoclinic crystal phase (m-ZrO_2_). The melting and sintering temperatures of the cubic and tetragonal crystal phases were above 1500 °C, which belongs to the high-temperature crystal phase. The presence of the high-temperature crystal phase of ZrO_2_ further proves that the plasma field exhibited a high-temperature environment, which provided conditions for the membrane melting and sintering and then improved the corrosion resistance of the ZrO_2_ ceramic membrane. The remaining XRD peaks were MgF_2_ peaks.

#### 3.7.2. PEO Membrane Corrosion Performance

**Figure 12:**
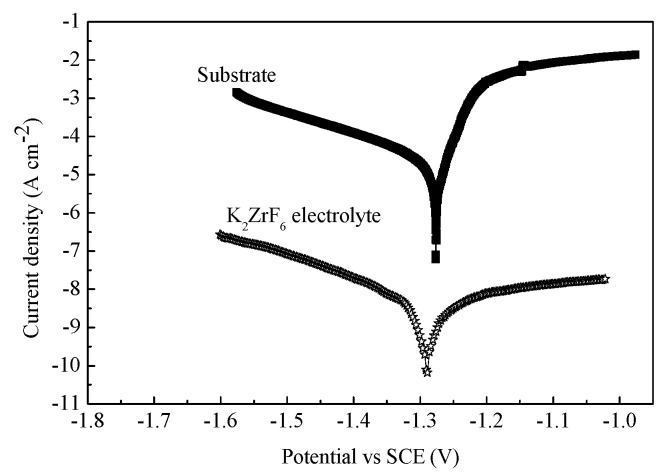
The potential dynamic curves of the ZrO_2_ ceramic membranes and the substrate.

Figure 12 shows the potentiodynamic polarization curve of the ZrO_2_ ceramic membrane. The polarization curve was fitted according to the tafel curve-fitting software provided by the electrochemical workstation. The corrosion potential of the ZrO_2_ ceramic membrane was −1.29 V, the corrosion current density was 3.1 × 10^−9^ (A/cm^2^), and the corrosion resistance was 6.5 × 10^7^ Ω. Compared to the substrate (corrosion potential was −1.64 V, corrosion current density was 6.3 × 10^−3^ (A/cm^2^), and corrosion resistance was 109.8 Ω), the corrosion current density of the PEO ceramic membrane was improved by six orders, which is attributed to the high-temperature phase formation of the ZrO_2_.

## 4. Conclusions

(1) The cations had the highest spectra intensity related to the lowest excitation energy with the higher plasma concentration. Although the anion of the electrolyte does not contribute to the composition of the plasma active species, it plays an important role in the charge balance of the entire system and the composition of the membrane layer. The intensity of active species during the PEO process is related to the energy state of the working electrode’s surface. The more energy there is, the more likely it is that the active species will be excited to generate energy level transitions.
(2) The heat and mass transfer during the PEO process were analyzed, and the PEO films’ growth mechanism was also proposed. The ion transfer at different stages exhibited different tendencies. At the conventional oxidation stage and transition stage, the migration resistance of the ions was low and increased gradually. At the initial discharge stage, the migration resistance was the highest because the highest membrane growth rate occurred at this stage. At the later discharge stage, the migration resistance tended to be stable, which is ascribed to a dynamic equilibrium PEO membrane growth rate.
(3) The prepared PEO ceramic membranes had a uniform surface with many different inner discharge channels distributed in the outside discharge channel. This proves that the PEO membranes grow layer by layer from the inner layer to the outer layer.
(4) The corrosion current density of the ZrO_2_ ceramic membrane was improved by six orders of magnitude compared with the AZ31B substrate, a result attributed to the high-temperature phase formation of the cubic, tetragonal, and monoclinic ZrO_2_.

## Figures and Tables

**Table 1:** List of the observed molecular bands, the corresponding transitions, and the wavelengths.

Species	λ (nm)	Transition	∆E(eV)
O_2_	686.7	$b^{1}\sum_{g}^{+}\left(v\prime =1\right)\to X^{3}\sum_{g}^{-}\left(v\prime \prime =0\right)$	-
O_2_	627.6	$b^{1}\sum_{g}^{+}\left(v\prime =2\right)\to X^{3}\sum_{g}^{-}\left(v\prime \prime =0\right)$	-
O_2_	538.0	$b^{1}\sum_{g}^{+}\left(v\prime =4\right)\to X^{3}\sum_{g}^{-}\left(v\prime \prime =0\right)$	-
O	847.0	$e+O_{2}(A^{3}\sum_{u}^{+})\to e+O{(}^{3}P)+O{(}^{1}D)$	-
OH	309.3	$A^{2}\sum_{}^{+}\left(v\prime =0\right)\to X^{2}\prod \left(v\prime \prime =0\right)$	-
OH	512.3	$B^{2}\sum_{}^{+}\left(v\prime =0\right)\to A^{2}\sum_{}^{+}\left(v\prime \prime =7\right)$	-
OH	882.9	$C^{2}\sum_{}^{+}\left(v\prime =7\right)\to X^{2}\prod \left(v\prime \prime =3\right)$	-
H_2_O	716.4	(3 0 1)–(0 0 0)	-
H_2_O^+^	659.4	(0 7 0)–(0 0 0)	-
O_2_^+^	383.0	$A^{2}\prod_{u}(v\prime =0)\to X^{2}\prod_{g}\left(v\prime \prime =8i\right)$	-
Na	589.0	3p-3s	2.101 [22]
K	769.9	4s-3d	2.650 [23]
Al	309.3	3d-3p	3.613 [24]
Mg	519.4	-	7.654 [24]

## Data Availability

Not applicable.
